# Treatment patterns and clinical profile in progressive pulmonary fibrosis: a Japanese cross-sectional survey

**DOI:** 10.3389/fmed.2024.1526531

**Published:** 2025-01-15

**Authors:** Hidekata Yasuoka, Yuko Waseda, Yuko Kaneko, Masateru Okazaki, Ryoko Iwasaki, Shoko Nagata, Mark Small, Haruyuki Ishii

**Affiliations:** ^1^Division of Rheumatology, Department of Internal Medicine, School of Medicine, Fujita Health University, Toyoake, Japan; ^2^Department of Respiratory Medicine, Faculty of Medical Sciences, University of Fukui, Eiheiji, Japan; ^3^Division of Rheumatology, Department of Internal Medicine, School of Medicine, Keio University, Tokyo, Japan; ^4^Nippon Boehringer Ingelheim Co., Ltd, Tokyo, Japan; ^5^Adelphi Real World, Bollington, United Kingdom; ^6^Department of Respiratory Medicine, Faculty of Medicine, Kyorin University, Tokyo, Japan

**Keywords:** idiopathic pulmonary fibrosis, progressive pulmonary fibrosis, interstitial lung disease, antifibrotics, treatment, real-world data

## Abstract

**Background:**

There is a paucity of real-world data on patients with interstitial lung diseases (ILDs) that are progressive, other than idiopathic pulmonary fibrosis (IPF), including treatment patterns and attitudes toward treatment. This study aimed to investigate the diagnosis, clinical characteristics, treatment paradigm and current decision-making practices of IPF and progressive pulmonary fibrosis (PPF) in a Japanese real-world setting.

**Methods:**

Data were drawn from the Adelphi Real World PPF-ILD Disease Specific Programme™, a cross-sectional survey with retrospective data collection of pulmonologists and rheumatologists in Japan from April to October 2022. Physicians provided data for up to 12 consecutive patients with a physician-confirmed diagnosis of progressive ILD; patients were also invited to complete patient self-completion forms. Analyses were descriptive.

**Results:**

A total of 63 physicians (43 pulmonologists and 20 rheumatologists) provided data on 312 patients with PPF and 70 patients with IPF. Patients had a mean (standard deviation [SD]) age at survey date of 68.0 (11.6) years, 43.5% were female, 50.3% were former smokers and 18.1% were employed full time. For breathlessness, 26.5% of patients had Grade 2 physician-reported breathlessness; this was 16.7% when reported by patients themselves. A total of 81.4% of patients were currently receiving treatment for ILD. Mean (SD) duration of current treatment was 1.5 (1.4) years. Slowing disease progression was the primary reason influencing physicians’ choice of current ILD treatment (48.5%). A total of 16.0% had never been treated (most frequent physician-reported reason: disease was manageable without treatment, 55.7%) and 2.6% had treatment discontinued (most frequent reason: patient request, 70.0%). Physicians reported 82.3% of patients as fully compliant with their treatment regimen. As reported by patients themselves (*n* = 53), 49.1% never and 37.7% rarely missed a dose.

**Conclusion:**

This analysis of real-world data from Japan provides insights into the clinical profile of patients with IPF and PPF in Japan, and highlights differences between physicians and patients in perception of symptom severity and attitudes to treatment.

## 1 Introduction

Idiopathic pulmonary fibrosis (IPF) is the archetypal progressive fibrosing interstitial lung disease (ILD) and is characterized by chronic progressive fibrosis, worsening of lung function and dyspnoea (1, 2). Approximately one third of patients with fibrosing ILDs other than IPF may experience disease progression (3, 4), defined as progressive pulmonary fibrosis (PPF) in the American Thoracic Society (ATS), the European Respiratory Society (ERS), the Japanese Respiratory Society (JRS), and the Latin American Thoracic Association (ALAT) 2022 guidelines (1).

PPF is characterized by worsening respiratory symptoms, decline in lung function, radiographic progression and early mortality despite appropriate management, and may have a clinical course similar to IPF (2, 5, 6). Regardless of the underlying condition, PPF occurs through similar mechanisms of self-sustained dysregulated cell repair, fibroblast proliferation and alveolar dysfunction (2, 6).

Both IPF and PPF are associated with high mortality. IPF has a median survival time of 3 to 5 years after diagnosis (7). In an analysis of placebo groups from clinical trials in patients with IPF and PPF, the 52-week mortality for IPF and PPF was similar (7.8% versus 5.1%, respectively) (6). In a Japanese patient cohort, the 5-year survival rate was 53.7% for patients with IPF and 72.8% for patients with PPF (8).

Both IPF and PPF are rare diseases, but the relative frequency of the different types of ILD varies, with sarcoidosis, connective tissue disease (CTD)-ILD and IPF the most common fibrotic ILDs (3). The estimated adjusted global incidence and prevalence for IPF in 2020 were 0.9–13.0 and 3.3–45.1 per 100,000 persons, respectively (9). The global incidence and prevalence of PPF are estimated at 2.1–32.6 per 100,000 person-years and 6.9–70.3 per 100,000 persons, respectively (10). There is limited epidemiological data available for Japan, particularly for patients with PPF. However, based on analyses of a medical claims database, the prevalence of IPF was estimated at 27.0 per 100,000 persons (11). A similar analysis of patients with systemic sclerosis (SSc)-ILD, found the overall incidence rate of SSc-ILD was 1.9 per 100,000 person-years and the overall prevalence was 13.9 per 100,000 persons.

While each of the individual fibrosing ILDs is rare, both IPF and PPF have a significant impact on patients in terms of symptom burden (9). And while there is a good understanding of the clinical characteristics and treatment paradigms of IPF (1), less is known about the burden of PPF (12), and there is a lack of understanding of the best approach to diagnose and treat PPF. While the ATS/ERS/JRS/ALAT 2022 guidelines have defined PPF and include a conditional recommendation for treatment of PPF in patients who have failed standard management for fibrotic ILD other than IPF (1), there is a lack of understanding of real-world treatment patterns and attitudes toward treatment. The Adelphi Real World PPF-ILD Disease Specific Programme (DSP)™ is a large, real-world, cross-sectional survey with elements of retrospective data collection of patients with ILD. This study aimed to investigate the diagnosis, clinical characteristics, treatment paradigm and current decision-making practices of PPF specifically in a Japanese real-world setting, by extracting and analyzing data from the Japanese cohort of the global DSP. As well as physician-reported clinical data, we include patient-reported attitudes to treatment. This study was devised before publication of the updated 2022 ATS/ERS/JRS/ALAT guidelines for IPF and PPF (1). However, for clarity, the term PPF is used throughout this manuscript.

## 2 Materials and methods

### 2.1 Data source

**FIGURE 1:**
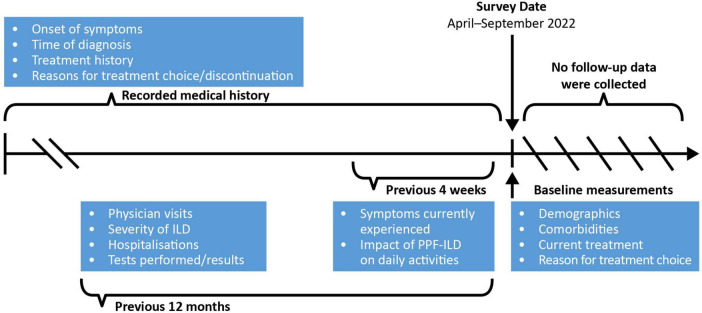
Schematic of information collection in the PPF-ILD DSP study. DSP, Disease Specific Programme; ILD, interstitial lung disease; IPF, idiopathic pulmonary fibrosis; PPF, progressive pulmonary fibrosis.

Data were drawn from the Japanese cohort of the Adelphi Real World PPF-ILD DSP™, which was conducted in Japan from April to September 2022. The DSP methodology has been previously described, validated and found to be representative and consistent over time (13–15). All information was recorded at a single point in time using available medical history or a specified recall period (4 weeks or 12 months depending on topic) for physicians (Figure 1); no follow-up information was collected.

Participating physicians reported data on patient demographics, clinical characteristics, symptom burden and impact, patient management, treatment utilization and decision-making in routine care. Physicians were instructed to complete patient record forms (PRFs) for up to 12 consecutively consulted patients with a physician-confirmed ILD diagnosis with, in their opinion, a progressive phenotype (10, 16) (this could include IPF). Each PRF included details regarding patient demographics, clinical characteristics (including physician-perceived disease severity, or according to Goh’s criteria (17) for SSc-ILD only, at different stages in the patient’s journey), symptom burden and treatment history. Physicians completed the PRF using a combination of existing patient clinical records, their own judgement and diagnostic/interpretation skills, and information collected during the consultation. Patients with a corresponding completed PRF were asked by their physician to complete a patient self-report form immediately after consultation on a voluntary basis. Patients provided information on demographics and their ILD journey. Physician- and patient-reported disease severity (mild, moderate, severe) and progression (improving, stable, progressing) were based on physician or patient opinion and not predefined. The terms “[progressive fibrosing] PF-ILD” and “[connective tissue disease] CTD-ILD” were used in the physician surveys and PRFs.

This study analyzed data from the Japanese population within the original cohort. Target physicians in Japan were pulmonologists and rheumatologists identified from public lists of healthcare professionals (HCPs). To be included in the survey, pulmonologists were required to see at least four different types of qualifying ILDs in a typical month, and rheumatologists were required to see at least two different types of CTD-ILD [rheumatoid arthritis (RA)-ILD, SSc-ILD, polymyositis/dermatomyositis-ILD or Sjögren’s-associated ILD] in a typical month. Patients were eligible for inclusion if they were aged over 18 years, had a physician-confirmed diagnosis of ILD and presented with a progressive phenotype, as determined by the reporting physician. No clinical definition of a progressive phenotype was pre-specified as the DSP survey was performed prior to publication of the current ATS/ERS/JRS/ALAT guidelines (1). Patients with the following diagnoses were included: IPF, CTD-ILD (SSc-ILD, RA-ILD, polymyositis/dermatomyositis-ILD and Sjögren’s-associated ILD) or other ILD (idiopathic non-specific interstitial pneumonia [iNSIP], fibrotic hypersensitivity pneumonitis and unclassifiable ILD). The patient journey and burden of IPF and PPF from both the physician and patient perspective are shown in an accompanying manuscript (submitted alongside the current manuscript) whereas the clinical characteristics and treatment profile of this cohort are reported here.

### 2.2 Data analyses

As the primary research objective was descriptive in nature (i.e., no *a priori* hypotheses specified), the available sample size of physicians and patients was driven by the DSP data collection methodology. Therefore, formal sample size calculations were not applicable and were not performed, and the sample size impacted the precision of any estimates. Descriptive analyses were undertaken by Adelphi Real World and conducted in UNICOM^®^ Data Collection Survey Reporter (UNICOM Global, Inc., Mission Hills, CA, USA). Descriptive statistics were used to characterize demographics, clinical characteristics, symptom burden/impact and treatment history.

### 2.3 Ethics statement

Data were collected by local fieldwork partners, and both physician and patient data were de-identified prior to receipt by Adelphi. The DSP received Pearl Institutional Review Board ethical exemption (exemption code #22-ADRW-135) and was conducted adhering to European Pharmaceutical Market Research Association international guidelines, of which Japan is a signatory.

## 3 Results

### 3.1 PPF-ILD DSP survey sample

Overall, 63 physicians reported on 382 patients, including 70 patients with IPF and 312 patients with a physician-confirmed ILD diagnosis with PPF (Table 1). The physicians were comprised of 43 pulmonologists and 20 rheumatologists. A total of 68 patients completed patient self-completion (PSC) forms.

**TABLE 1:** PPF-ILD DSP sample size.

		Physician specialty	
	**Total**	**Pulmonologists**	**Rheumatologists**
Physician surveys	63	43	20
Patient record forms	382	260	122
Patient self-report form	68	49	19

DSP, Disease Specific Programme; ILD, interstitial lung disease; PPF, progressive pulmonary fibrosis.

### 3.2 Patient demographics

The mean (standard deviation [SD]) age of patients (*n* = 382) at survey date was 71.9 (7.3) years for patients with IPF, 64.1 (12.6) years for patients with CTD-ILD and 70.6 (10.8) years for patients with other ILDs (Table 2). Overall, patients were 43.5% female, 50.3% were former smokers, 4.2% were current smokers, and mean (SD) body mass index was 22.1 (3.1) kg/m^2^. Patient sex differed by diagnosis; female patients comprised 12.9% of patients with IPF, 65.3% of patients with CTD-ILDs and 33.1% of patients with other ILDs. Few patients (4.5%) had a family history of ILD. At the survey date, a total of 18.1% of patients were in full-time employment. Of those not in full-time employment (*n* = 214), 3.3% were unable to work full time due to their ILD (either working part time, retired, on long-term sick leave or unemployed).

**TABLE 2:** Patient demographics.

			Type of PPF	
	**Total population (*n* = 382)**	**IPF (*n* = 70)**	**CTD-ILD (*n* = 167)**	**Other ILDs (*n* = 145)**
Age, mean (SD)	68.0 (11.6)	71.9 (7.3)	64.1 (12.6)	70.6 (10.8)
BMI, mean (SD)	22.1 (3.1)	21.9 (2.4)	21.8 (3.2)	22.6 (3.2)
Female sex, *n* (%)	166 (43.5)	9 (12.9)	109 (65.3)	48 (33.1)
**Ethnicity, *n* (%)**				
Japanese	380 (99.5)	70 (100.0)	165 (98.8)	145 (100.0)
Korean	2 (0.5)	0 (0.0)	2 (1.2)	0 (0.0)
**Smoking status, *n* (%)**				
Current smoker	16 (4.2)	5 (7.1)	5 (3.0)	6 (4.2)
Ex-smoker	192 (50.3)	55 (78.6)	53 (31.7)	84 (57.9)
Never smoked	145 (37.9)	7 (10.0)	91 (54.5)	47 (32.4)
Don’t know	29 (7.6)	3 (4.3)	18 (10.8)	8 (5.5)
**Employment status, *n* (%)**				
Working full time	69 (18.1)	9 (12.9)	44 (26.3%)	16 (11.0)
Working part time	26 (6.8)	4 (5.7)	11 (6.6%)	11 (7.6)
On long-term sick leave	2 (0.5)	1 (1.4)	1 (0.6%)	0 (0.0)
Homemaker	87 (22.8)	6 (8.6)	62 (37.1)	19 (13.1)
Retired	111 (29.1)	30 (42.8)	26 (15.6)	55 (37.9)
Unemployed	75 (19.6)	17 (24.3)	19 (11.4)	39 (26.9)
Unknown	12 (3.1)	3 (4.3)	4 (2.4)	5 (3.5)
**Diagnosis, *n* (%)**				
IPF	70 (18.3)	70 (100)	NA	NA
iNSIP	83 (21.7)	NA	NA	83 (57.2)
fHP	37 (9.7)	NA	NA	37 (25.5)
uILD	25 (6.5)	NA	NA	25 (17.2)
SSc-ILD	49 (12.8)	NA	49 (29.3)	NA
RA-ILD	62 (16.2)	NA	62 (37.1)	NA
PM/DM-ILD	37 (9.7)	NA	37 (22.2)	NA
SS-ILD	19 (5.0)	NA	19 (11.4)	NA

BMI, body mass index; CTD, connective tissue disease; DM, dermatomyositis; fHP, fibrotic hypersensitivity pneumonitis; ILD, interstitial lung disease; iNSIP, idiopathic non-specific interstitial pneumonia; IPF, idiopathic pulmonary fibrosis; NA, not applicable; PM, polymyositis; PPF, progressive pulmonary fibrosis; RA, rheumatoid arthritis; SD, standard deviation; SS, Sjögren’s; SSc, systemic sclerosis; uILD, unclassifiable interstitial lung disease.

### 3.3 Clinical characteristics

**FIGURE 2:**
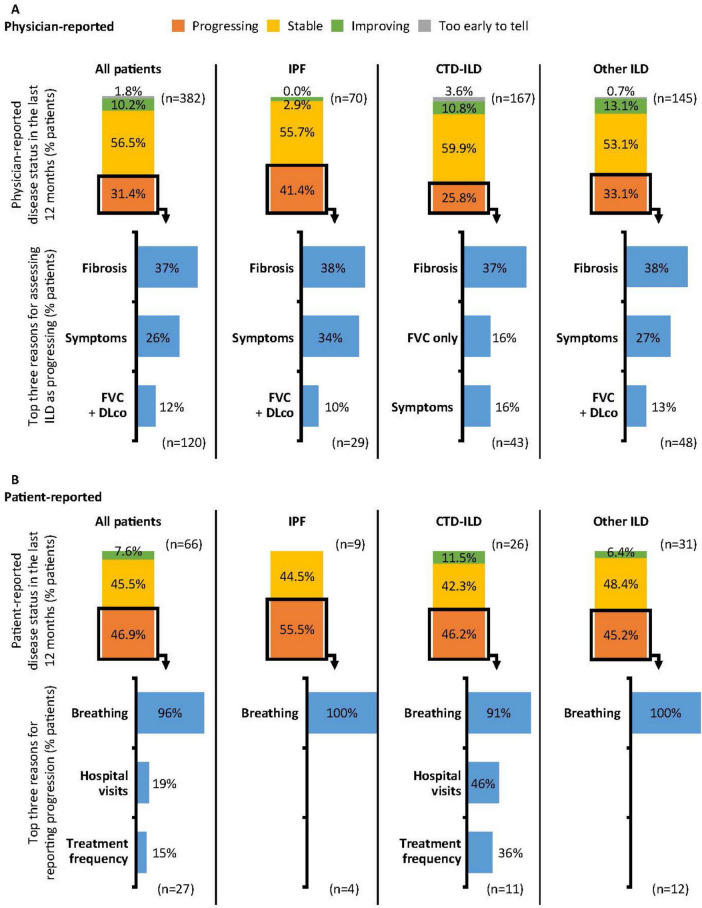
Physician- **(A)** and patient-reported **(B)** progression in the 12 months prior to survey date. Physician- and patient-reported disease progression was based on physician or patient opinion and not predefined. Reasons for reporting progression (physicians): Fibrosis: Increased fibrosis of the lungs as demonstrated by CT/HRCT scan; Symptoms: Increase in symptom severity; FVC + DLco: Decline in both FVC and DLco; FVC only: Decline in FVC only. Reasons for reporting progression (patients): Breathing: Due to my lung/breathing condition symptoms worsening; Hospital visits: Increased number of hospital visits; Treatment frequency: Increased frequency of treatment. CT, computed tomography; CTD, connective tissue disease; DLco, diffusing capacity of the lungs for carbon monoxide; FVC, forced vital capacity; HRCT, high-resolution computed tomography; ILD, interstitial lung disease; IPF, idiopathic pulmonary fibrosis.

Physician-reported (*n* = 382) disease severity at survey date was 48.2% mild, 45.0% moderate and 6.8% severe (Supplementary Figure 1). Patient-reported (*n* = 66) disease severity at survey date was 54.5% mild, 36.4% moderate and 9.1% severe (Supplementary Figure 1). Disease was extensive in 47.1% of patients and limited in 52.9% at the survey date, according to Goh’s criteria (17). Extensive disease was reported in a higher proportion of patients with IPF (58.6%) than CTD-ILD (38.9%), or non-CTD-ILD (51.0%). Physician-reported (*n* = 382) progression over the last 12 months prior to survey date was reported in 31.4% of patients. Progression differed between different types of ILD (Figure 2A). The most common reasons for physician-reported progression were increased fibrosis on high-resolution computed tomography (HRCT) (36.7%), symptom severity (25.8%), decline in forced vital capacity (FVC) and diffusing capacity of the lungs for carbon monoxide (DLco) (11.7%) and decline in FVC only (11.7%); this trend was consistent across ILD type.

**FIGURE 3:**
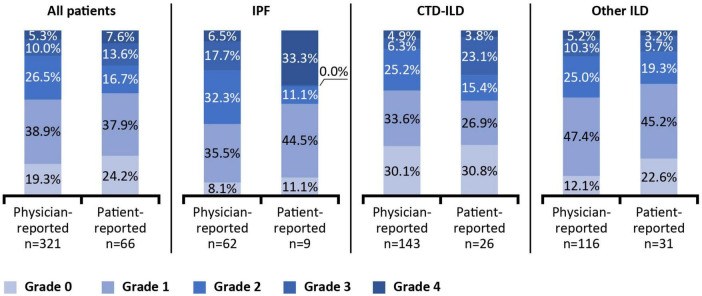
Physician- and patient-reported breathlessness at survey date. Breathlessness reported according to the modified Medical Research Council Dyspnoea Scale. Grade 0: Only gets breathless after strenuous exercise. Grade 1: Gets breathless when hurrying on level ground or walking up a slight incline. Grade 2: On level ground, walks slower than people of the same age because of breathlessness, or has to stop for breath when walking at own pace. Grade 3: Stops for breath after walking a few minutes on level ground. Grade 4: Is too breathless to leave the house or becomes breathless when getting dressed. CTD, connective tissue disease; ILD, interstitial lung disease; IPF, idiopathic pulmonary fibrosis.

As self-reported by patients (*n* = 66) for the 12 months prior to survey date, 46.9% experienced their ILD getting worse and 45.5% reported their ILD as stable (Figure 2B). The most common reason for patients reporting their ILD as getting worse was due to worsening symptoms (96.3%). For functional impairment related to breathlessness, 26.5% of all patients were reported by physicians (*n* = 321) as walking slower than contemporaries on level ground because of breathlessness or having to stop for breath when walking at own pace (Grade 2) at the survey date (Figure 3), 10.0% stop for breath after walking a few minutes on level ground (Grade 3), and were breathless to leave the house or become breathless when getting dressed (Grade 4). When reported by patients (*n* = 66), 16.7% experienced Grade 2 breathlessness, 13.6% experienced Grade 3 and 7.6% experienced Grade 4.

Breathlessness varied by type of ILD for both physician- and patient-reported measures, and there were reporting discrepancies across the grades. Physicians reported 5.3% of all patients having Grade 4 breathlessness (too breathless to leave the house or breathless when dressing or undressing), whereas 33.3% of patients with IPF self-reported as having Grade 4 breathlessness; however, this was based on data from only 66 patients overall and 9 with IPF.

### 3.4 Treatment

#### 3.4.1 Treatment profile

At survey date, 81.4% of patients were receiving treatment for their ILD, 2.6% had previously been prescribed treatment for ILD but were not currently being treated, and 16.0% had never been treated for their ILD (Table 3). The mean (SD) duration of current treatment was 1.5 (1.4) years. The current prescribing physician was a pulmonologist or respiratory specialist for 72.0% of patients, and a rheumatologist for 32.5% of patients (Table 3). For patients with CTD-ILD (*n* = 137), a rheumatologist was the prescribing physician for 73.0% of patients.

**TABLE 3:** Current treatment patterns.

			Type of PPF	
	**All patients**	**IPF**	**CTD-ILD**	**Other ILD**
**Currently receiving treatment for ILD, *n* (%)**	**382**	**70**	**167**	**145**
Yes	311 (81.4)	61 (87.2)	137 (82.0)	113 (77.9)
No, but previously prescribed treatment	10 (2.6)	1 (1.4)	6 (3.6)	3 (2.1)
No, has never been prescribed treatment	61 (16.0)	8 (11.4)	24 (14.4)	29 (20.0)
**Current treatment regimen^1^ ≥ 5%, *n* (%)**	**311**	**61**	**137**	**113**
Nintedanib	152 (48.9)	47 (77.0)	59 (43.1)	46 (40.7)
Low-dose prednisone	121 (38.9)	12 (19.7)	59 (43.1)	50 (44.2)
Cough suppressants	69 (22.2)	15 (24.6)	22 (16.1)	32 (28.3)
Tacrolimus	51 (16.4)	1 (1.6)	46 (33.6)	4 (3.5)
High-dose prednisone	37 (11.9)	0 (0.0)	21 (15.3)	16 (14.2)
Oxygen therapy	26 (8.4)	7 (11.5)	11 (8.0)	8 (7.1)
Mycophenolate	18 (5.8)	0 (0.0)	18 (13.1)	0 (0.0)
Pirfenidone	17 (5.5)	8 (13.1)	2 (1.5)	7 (6.2)
Tocilizumab	17 (5.5)	1 (1.6)	13 (9.5)	3 (2.7)
**Current line of treatment for ILD, *n* (%)**	**305**	**60**	**133**	**112**
First line	250 (82.0)	46 (76.7)	106 (79.7)	98 (87.5)
Nintedanib	114 (45.6)	37 (80.4)	40 (37.7)	37 (37.8)
Low-dose prednisone	91 (36.4)	5 (10.9)	44 (41.5)	42 (42.9)
Cough suppressants	53 (21.2)	10 (21.7)	18 (17.0)	25 (25.5)
Tacrolimus	39 (15.6)	1 (2.2)	34 (32.1)	4 (4.1)
High-dose prednisone	31 (12.4)	0 (0)	19 (17.9)	12 (12.2)
Oxygen therapy	14 (5.6)	3 (6.5)	8 (7.5)	3 (3.1)
Second line	41 (13.4)	10 (16.7)	21 (15.8)	10 (8.9)
Third line	11 (3.6)	4 (6.6)	3 (2.2)	4 (3.6)
Fourth line	3 (1.0)	0 (0.0)	3 (2.3)	0 (0.0)

^1^Multiple answers could be selected in response to this question. CTD, connective tissue disease; ILD, interstitial lung disease; IPF, idiopathic pulmonary fibrosis; PPF, progressive pulmonary fibrosis.

For patients receiving treatment at survey date, the most common treatments for patients with IPF (*n* = 61) were nintedanib (77.1%), cough suppressants (24.6%) and low-dose prednisone (19.7%) (Table 3). For patients with CTD-ILD (*n* = 137), the most common treatments were nintedanib (43.1%), low-dose prednisone (43.1%) and tacrolimus (33.6%). For patients with other ILDs (*n* = 113), the most common treatments were low-dose prednisone (44.3%), nintedanib (40.7%) and cough suppressants (28.3%).

For those patients with CTD-ILD (*n* = 137), the most frequently prescribed treatments for their underlying autoimmune condition were corticosteroids (36.5%) and immunosuppressants (34.3%) (Supplementary Table 1).

Overall, 82.0% of patients were prescribed first-line treatment for ILD at survey date (Table 3). The most frequent first-line treatments were nintedanib (45.6%) and low-dose prednisone (36.4%). First-line treatment differed by type of ILD, with 80.4% of these patients with IPF receiving nintedanib as first-line treatment, with 41.5% and 42.9% of these patients with CTD-ILD or other ILDs, respectively, receiving low-dose prednisone as first-line treatment.

#### 3.4.2 Treatment decisions

Slowing disease progression was the primary reason that influenced physicians’ choice of current ILD treatment (48.5%) (Table 4) and was consistent for patients with CTD-ILD (53.7%) and IPF (52.5%). However, for patients with other ILDs, improving cough was the primary reason for choice of ILD treatment (58.9%). Slowing disease progression (36.6%) and improving dyspnoea (30.7%) were identified by physicians as the most important areas of improvement that would be of benefit to the patient with their current treatment (Supplementary Table 3).

**TABLE 4:** Treatment decisions.

			Type of PPF	
	**All patients**	**IPF**	**CTD-ILD**	**Non-CTD ILD**
**Primary reason of choice for current treatment for ILD ≥ 10%, *n* (%)**	**309**	**61**	**136**	**112**
Slows disease progression	150 (48.5)	32 (52.5)	73 (53.7)	45 (40.2)
Improves dyspnoea	142 (46.0)	20 (32.8)	69 (50.7)	53 (47.3)
Improves cough	128 (41.4)	20 (32.8)	42 (30.9)	66 (58.9)
Improves patients’ long-term outcomes	120 (38.8)	29 (47.5)	56 (41.2)	35 (31.3)
Reduced frequency of acute exacerbations of ILD	106 (34.3)	29 (47.5)	45 (33.1)	32 (28.6)
Physician familiarity/experience	93 (30.1)	16 (26.2)	40 (29.4)	37 (33.0)
Used in accordance with guidelines	93 (30.1)	22 (36.1)	46 (33.8)	25 (22.3)
Improves survival/reduces ILD-related mortality	92 (29.8)	17 (27.9)	48 (35.3)	27 (24.1)
Maintains efficacy over time	90 (29.1)	19 (31.1)	37 (27.2)	34 (30.4)
Safe long-term use	86 (27.8)	16 (26.2)	39 (28.7)	31 (27.7)
On formulary/hospital-approved drug list	63 (20.4)	12 (19.7)	35 (25.7)	16 (14.3)
Enables patients to engage in more activities of daily living	49 (15.9)	4 (6.6)	19 (14.0)	26 (23.2)
Demonstrates a manageable overall safety profile	49 (15.9)	8 (13.1)	22 (16.2)	19 (17.0)
Dosing schedule convenience	47 (15.2)	9 (14.8)	16 (11.8)	22 (19.6)
Method of administration is acceptable to patients	40 (12.9)	7 (11.5)	18 (13.2)	15 (13.4)
Improves fatigue	39 (12.6)	6 (9.8)	23 (16.9)	10 (8.9)
Can be used continuously (without a drug break)	35 (11.3)	11 (18.0)	8 (5.9)	16 (14.3)
Good side effect profile	31 (10.0)	6 (9.8)	16 (11.8)	9 (8.0)
**Reasons for never prescribing treatment ≥ 5, *n* (%)**	**61**	**8**	**24**	**29**
Profile is manageable without treatment	34 (55.7%)	0 (0%)	18 (75%)	16 (55.2%)
Patient request	18 (29.5)	5 (62.5)	2 (8.3)	11 (37.9)
Patient concerns over side effects	9 (14.8)	0 (0.0)	2 (8.3)	7 (24.1)
Symptoms not severe enough to warrant treatment	9 (14.8)	1 (12.5)	2 (8.3)	6 (20.7)
Diagnosed too recently	7 (11.5)	2 (25.0)	1 (4.2)	4 (13.8)
**Reason for discontinuation of ILD treatment ≥ 10, *n* (%)**	**10**	**1**	**6**	**3**
Patient request	7 (70.0)	1 (100.0)	3 (50.0)	3 (100.0)
Poor adherence to prescribed treatment	2 (20.0)	1 (100.0)	0 (0.0)	1 (33.3)
Decline in health-related quality of life	2 (20.0)	0 (0.0)	1 (16.7)	1 (33.3)
Due to the number of side effects experienced	2 (20.0)	0 (0.0)	1 (16.7)	1 (33.3)
Due to the severity of side effects experienced	2 (20.0)	0 (0.0)	1 (16.7)	1 (33.3)
ILD symptoms worsened	1 (10.0)	0 (0.0)	1 (16.7)	0 (0.0)
Cost of medication	1 (10.0)	0 (0.0)	1 (16.7)	0 (0.0)
Other	2 (20.0)	0 (0.0)	1 (16.7)	1 (33.3)
**Next course of action for inadequate response ≥ 5, *n* (%)**	**311**	**61**	**137**	**113**
Increase dose	97 (31.2%)	10 (16.4%)	45 (32.8%)	42 (37.2%)
Add on a product to current regimen	77 (24.8%)	11 (18%)	48 (35%)	18 (15.9%)
Oxygen support	62 (19.9)	19 (31.2)	20 (14.6)	23 (20.3)
Pulmonary rehabilitation	25 (8.0)	8 (13.1)	4 (2.9)	13 (11.5)
Switch product and replace	18 (5.8)	3 (4.9)	10 (7.3)	5 (4.4)
Don’t know	24 (7.7)	9 (14.8)	6 (4.4)	9 (8.0)

CTD, connective tissue disease; ILD, interstitial lung disease; IPF, idiopathic pulmonary fibrosis; PPF, progressive pulmonary fibrosis.

The most frequent reason for never prescribing ILD treatment was that the disease was manageable without treatment (55.7%), and the most frequent physician-reported reason for discontinuation of ILD treatment was at the patients’ request (70.0%) (Table 4). If the patient’s current treatment proved inadequate, physicians reported (*n* = 311) that their main next course of action would be to increase dose (31.2%) or to add on a product to current regimen (24.8%) (Table 4). For patients with IPF (*n* = 61), 14.8% of physicians reported they could not say at this time what the next course of action would be following an inadequate response (Table 4).

For patients with IPF for whom the physicians would increase the dose (*n* = 10), 70.0% would increase the dose of nintedanib (Supplementary Table 2). Where the physicians would add on to the current regimen (*n* = 11), 45.5% would add on pirfenidone and 27.3% would add on nintedanib.

For patients with CTD-ILD for whom the physicians would increase the dose (*n* = 45), 44.4% would increase the dose of low-dose prednisone (starting dose not reported), 33.3% would increase the dose of nintedanib, and 24.4% would increase the dose of tacrolimus (Supplementary Table 2). Where the physicians that would add on to the current regimen (*n* = 48), 52.1% would add on nintedanib and 16.7% would add cyclophosphamide.

For patients with other ILDs for whom the physicians would increase the dose (*n* = 42), 57.1% would increase the dose of low-dose prednisone (starting dose not reported) and 19.1% would increase the dose of high-dose prednisone (Supplementary Table 2). Where the physicians that would add on to the current regimen (*n* = 18), 44.4% would add on nintedanib, and 27.8% would add on low-dose prednisone.

#### 3.4.3 Treatment adherence and satisfaction

Physician-reported patient adherence to current treatment (*n* = 311) was 41.8% completely adherent and 52.1% mostly adherent, and 82.3% of patients were rated as fully compliant with their treatment regimen (patient took > 80% of prescribed dose) (Table 5). The main reasons reported by physicians for their patients not taking their medication at times during the 12 months prior to survey date (*n* = 18) were that the patient did not see an improvement and the patient did not feel instant results (both 38.9%) (Supplementary Table 4). As reported by patients themselves (*n* = 53), the most frequent response was that they never or rarely missed a dose (49.1 and 37.7%, respectively) and never or rarely took their medication at a different time than advised by their doctor (41.5% and 37.7%, respectively) (Supplementary Table 5). Most patients also never or rarely took more (71.7% and 24.5%) or less (66.0% and 26.4%) than their recommended dose.

**TABLE 5:** Physician-reported treatment adherence.

			Type of PPF	
	**All patients**	**IPF**	**CTD-ILD**	**Other ILD**
**Patient adherence to current ILD treatment, *n* (%)**	**311**	**61**	**137**	**113**
Completely adherent	130 (41.8)	24 (39.4)	50 (36.5)	56 (49.6)
Mostly adherent	162 (52.1)	35 (57.4)	75 (54.8)	52 (46.0)
Somewhat adherent	14 (4.5)	1 (1.6)	9 (6.6)	4 (3.5)
A little adherent	3 (1.0)	1 (1.6)	1 (0.7)	1 (0.9)
Not at all adherent	1 (0.3)	0 (0.0)	1 (0.7)	0 (0.0)
Too early in patient’s regimen to tell	1 (0.3)	0 (0.0)	1 (0.7)	0 (0.0)
**Patient compliance with treatment regimen, *n* (%)**	**311**	**61**	**137**	**113**
Fully compliant (takes > 80 of prescribed dose)	256 (82.3)	55 (90.2)	106 (77.4)	95 (84.1)
Fairly compliant (takes 50–80 of prescribed dose)	35 (11.3)	3 (4.9)	18 (13.1)	14 (12.4)
Poor compliance (takes < 50 of prescribed dose)	7 (2.2)	2 (3.3)	4 (2.9)	1 (0.9)
Too early in patient’s regimen to tell	7 (2.3)	0 (0.0)	7 (5.1)	0 (0.0)
Not at all—does fill prescriptions but does not take them	5 (1.6)	1 (1.6)	2 (1.5)	2 (1.7)
Not at all—does not fill prescriptions	1 (0.3)	0 (0.0)	0 (0.0)	1 (0.9)

CTD, connective tissue disease; ILD, interstitial lung disease; IPF, idiopathic pulmonary fibrosis; PPF, progressive pulmonary fibrosis.

## 4 Discussion

The current study provides real-world insights into the clinical profile of patients with IPF and PPF in Japan, addressing the paucity of data regarding clinical characteristics and treatment patterns in these patients. Data from the study were discussed with patients during two advisory boards (Supplementary Data Sheets 1, 2), and some of their insights are included here.

Disease progression and severity were not defined in the survey and were classified as perceived by physicians. This study was devised before publication of the ATS/ERS/JRS/ALAT 2022 guidelines; however, patients were required to have an ILD that the physician considered to be a progressive phenotype. The definitions for disease status were purposefully left open to gain physician judgement of what counts as progression or stability. This method is representative of physicians’ real-world classification of patients and has been used in previous publications of the DSP (18). In addition, approximately half of the patients in all groups showed progression of subjective symptoms despite 81% of patients being on treatment. Progression was reported more frequently in patients with IPF than other forms of ILD; this may reflect the impact of anti-inflammatory treatment on perceived progression. Thirty-one percent of physicians and 47% of patients self-reported progressing over the 12 months prior to the survey date, highlighting the continuing unmet need for effective and timely treatment of IPF and PPF. Physicians use objective measures to assess progression, whereas patients rely on how they feel. In the patient advisory boards, patients perceived that they did not receive sufficient explanation from their doctors about their disease status, including its severity. Additionally, as test results did not always match their symptoms, patients found it difficult to get their doctor to understand their condition and were not satisfied with their treatment. To increase patient understanding of their disease and satisfaction with treatment, a continued dialogue with the patient is important, in addition to having, and fully explaining, test results. Physicians may prioritize objective imaging tests HRCT and pulmonary function tests in assessing the disease and its severity, but our findings highlight the need to listen to the patients’ own assessment of their symptoms to improve the gap in perception between patient and physician. Increased awareness and use of instruments such as the King’s Brief Interstitial Lung Disease (K-BILD) questionnaire (19) during consultations could help achieve this in clinical practice. Patients in the DSP were asked to complete the K-BILD instrument, and the results of these and other health-related quality of life instruments are reported in a separate manuscript.

The number of patients recorded as not working full time due to their ILD indicates the burden of disease for patients with IPF, CTD-ILD and other types of PPF. However, given the average patient age was 68 years, it could be expected that fewer patients were in full-time employment (just under 30% were retired). In addition, CTDs are systemic diseases that can affect various organs; therefore, some patients may be unable to work due to reasons other than their ILD. During the patient advisory board, participants mentioned that due to the progressive nature of IPF and PPF, they have had difficulties discussing their condition with their employer and whether they might need to change careers following a diagnosis; patients also referred to the “burden” on their colleagues if they are unable to work as normal. Patients said they needed support and options to better balance work and medical needs.

The most common reason patients were never prescribed treatment for fibrosing ILD was that fibrosing ILD was judged to be manageable without treatment. There was also a higher proportion of patients with CTD-ILD or other ILD who had never received treatment for ILD than for patients with IPF, although the reason for not prescribing treatment was not captured. This may indicate an unmet need for physician education on timely treatment of IPF and PPF to improve patient prognosis.

In terms of symptom impact, approximately a quarter of patients were reported as having breathlessness that made them slower than others of a similar age without ILD (Grade 2), with another 15% of patients experiencing even more severe breathlessness.

There was still a proportion of patients untreated despite diagnosis with progressive disease with high mortality rate and despite established treatment guidelines for IPF. For nearly 15% of patients with IPF, the choice over the next course of action was unknown for physicians following an inadequate response to treatment as there are limited treatment options after antifibrotic therapy. More treatment options are needed to add to the standard of care for both IPF and PPF. While 31% and 25% of physicians responded that their next course of action would be to increase the dose or to add on additional treatments to the current regimen, there are limits to how much this can be done. However, the survey did not address whether treatment changes would be due to ILD alone or for the underlying disease for patients with CTD-ILD. Treatment adherence was high in this study, as rated by both physicians and patients.

The current study has various limitations, including the small number of patients in some categories, limiting the scope of the conclusions. Participating patients may not reflect the general population of patients with PPF with other underlying ILDs. The study population may be skewed toward those more willing to consult physicians, or with more severe disease and undergoing monitoring for treatment response. Some of the patient-reported data, such as onset of symptoms, relied on patients’ memory, which may be affected by recall bias, and the timing of symptoms and their severity may be unknown. The use of real-time tracking and electronic diaries could ameliorate this in future studies. The study was not based on a true random sample of physicians or patients, as no formal patient selection verification procedures were in place and relied on physician judgement. However, to minimize selection bias, the sample comprised consecutive eligible patients, and all patients who met the eligibility criteria were included in the study. In addition, the first-line treatment might not be the first-line treatment of ILD as previous treatment history was not collected in the survey. Patients were included based on physicians’ subjective assessments of progression and so, it is possible that some patients who would not have met objective criteria for progression as proposed in current guidelines were included. Use of objective criteria may have allowed a more accurate picture of patients with PPF, their disease severity and treatment status.

The findings here relate to the treatment patterns and clinical profile in Japan only, but are broadly consistent with those of a larger DSP involving 265 physicians and 1,335 patients with PPF (in this case excluding IPF) in Europe and the USA (20). Approximately 16% of patients had never been treated in both studies, although if patients with IPF are excluded, this rises to around 21% for patients in Japan. Approximately 30% of patients in the Europe/USA study were prescribed nintedanib, compared with 40% of patients in Japan (excluding patients with IPF).

In conclusion, this analysis of real-world data from Japan provides insights into the clinical profile of patients with IPF and PPF in Japan, and highlights differences between physicians and patients in perception of symptom severity and attitudes to treatment, with different practices and treatment paradigms for IPF versus PPF. With PPF now defined in guidelines with recommendations for treatment, it will be interesting to observe how the clinical profile and treatment paradigm for IPF and PPF will change over time.

## Data Availability

The data analyzed in this study was subject to the following licenses/restrictions: Data are available from the authors upon reasonable request and with permission of Adelphi Real World. Requests to access these datasets should be directed to MS, marksmall@adelphigroup.com.
