# Supplementary material for: Treatment patterns and clinical profile in progressive pulmonary fibrosis: a Japanese cross-sectional survey
Source: Front Med (Lausanne). 2025 Jan 15;11:1526531. doi: 10.3389/fmed.2024.1526531 (PMC11775758; doi:10.3389/fmed.2024.1526531)
Supplement: Supplementary file 1 [file Table_1.docx]

Supplementary Material

# Supplementary Tables

**Supplementary Table S1.** Current treatment prescribed to treat underlying autoimmune condition

| **Treatment, %** | **CTD-ILD (n=137)** |
| --- | --- |
| Corticosteroids | 36.5 |
| Immunosuppressants (e.g. prednisone) | 34.3 |
| Conventional synthetic DMARD (e.g. leflunomide, methotrexate) | 30.7 |
| Antacids or proton pump inhibitors | 13.1 |
| Biologic or biosimilar DMARD (e.g. rituximab, tocilizumab, abatacept) | 10.9 |
| Non-steroidal anti-inflammatory drugs | 8.0 |
| Secretagogues (e.g. pilocarpine, cevimeline) | 5.1 |
| Endothelin receptor antagonist (e.g. ambrisentan) | 5.1 |
| Targeted synthetic DMARD (e.g. tofacitinib, baricitinib, apremilast) | 3.6 |
| Phosphodiesterase-5 inhibitors (e.g. sildenafil, tadalafil) | 3.6 |
| ACE inhibitors | 3.6 |
| Prostacyclin pathway agents (e.g. epoprostenol, treprostinol, iloprost) | 2.9 |
| B-blockers | 2.9 |
| Statins | 2.9 |
| Antidepressants | 2.2 |
| Other biologic (e.g. belimumab) | 1.5 |
| Anti-malarial drugs (e.g. hydrochloroquine) | 1.5 |
| Anticoagulants | 1.5 |
| Antibiotics (e.g. ciprofloxacin, metronidazole) | 0.7 |
| Phosphodiesterase-4 inhibitors (e.g. apremilast) | 0.7 |
| Other | 13.8 |
| Patient not receiving treatment for their CTD-ILD | 7.3 |

ACE, angiotensin converting enzyme; CTD, connective tissue disease; DMARD, disease-modifying antirheumatic drugs; ILD, interstitial lung disease.

**Supplementary Table S2.** Next course of action for ILD treatment

|  |  |  | **Type of PPF** | |
| --- | --- | --- | --- | --- |
|  | **All patients** | **IPF** | **CTD-ILD** | **Other ILD** |
| **Increase dose of ILD treatment, n (%)** | **97** | **10** | **45** | **42** |
| Low dose prednisone | 46.4 | 10.0 | 44.4 | 57.1 |
| Nintedanib | 24.7 | 70.0 | 33.3 | 4.8 |
| Tacrolimus | 11.3 | 0.0 | 24.4 | 0.0 |
| High dose prednisone | 11.3 | 0.0 | 6.7 | 19.1 |
| Cough suppressants | 8.3 | 10.0 | 6.7 | 9.5 |
| Tocilizumab | 5.2 | 10.0 | 4.4 | 4.8 |
| Mycophenolate | 4.1 | 0.0 | 8.9 | 0.0 |
| Pirfenidone | 3.1 | 10.0 | 0.0 | 4.8 |
| Azathioprine | 2.1 | 0.0 | 4.4 | 0.0 |
| Methotrexate | 2.1 | 0.0 | 4.4 | 0.0 |
| Cyclosporine | 1.0 | 0.0 | 2.2 | 0.0 |
| Inhaled corticosteroids | 1.0 | 0.0 | 2.2 | 0.0 |
| Upadacitinib | 1.0 | 0.0 | 2.2 | 0.0 |
| **Add on to current regimen, n (%)** | **77** | **11** | **48** | **18** |
| Nintedanib | 46.8 | 27.3 | 52.1 | 44.4 |
| Low dose prednisone | 14.3 | 18.2 | 8.3 | 27.8 |
| Pirfenidone | 11.7 | 45.5 | 2.1 | 16.7 |
| Cyclophosphamide | 10.4 | 0.0 | 16.7 | 0.0 |
| Oxygen therapy | 9.1 | 0.0 | 14.6 | 0.0 |
| Tacrolimus | 6.5 | 0.0 | 8.3 | 5.6 |
| High dose prednisone | 6.5 | 9.1 | 8.3 | 0.0 |
| Cyclosporine | 5.2 | 9.1 | 4.2 | 5.6 |
| Tocilizumab | 5.2 | 0.0 | 8.3 | 0.0 |
| Cough suppressants | 3.9 | 9.1 | 4.2 | 0.0 |
| Azathioprine | 2.6 | 0.0 | 4.2 | 0.0 |
| Rituximab | 2.6 | 0.0 | 4.2 | 0.0 |
| Generic pirfenidone | 1.3 | 0.0 | 0.0 | 5.6 |
| Mycophenolate | 1.3 | 0.0 | 2.1 | 0.0 |
| Inhaled corticosteroids | 1.3 | 0.0 | 2.1 | 0.0 |
| Apremilast | 1.3 | 0.0 | 2.1 | 0.0 |
| **Switch product, n (%)** | **18** | **3** | **10** | **5** |
| Switch nintedanib for pirfenidone | 27.8 | 100.0 | 30.0 | 20.0 |
| Switch tocilizumab for rituximab | 11.1 | 0.0 | 10.0 | 20.0 |
| Switch cough suppressants for nintedanib + low dose prednisone | 5.6 | 0.0 | 0.0 | 20.0 |
| Switch cough suppressants for pirfenidone | 5.6 | 0.0 | 0.0 | 0.0 |
| Switch cyclophosphamide for nintedanib + tocilizumab + rituximab | 5.6 | 0.0 | 10.0 | 0.0 |
| Switch low dose prednisone for high dose prednisone | 5.6 | 0.0 | 10.0 | 0.0 |
| Switch nintedanib for pirfenidone + low dose prednisone | 5.6 | 0.0 | 0.0 | 20.0 |
| Switch nintedanib for pirfenidone + oxygen therapy | 5.6 | 0.0 | 0.0 | 20.0 |
| Switch "other" for mycophenolate | 5.6 | 0.0 | 10.0 | 0.0 |
| Switch pirfenidone for nintedanib + cough suppressants | 5.6 | 0.0 | 10.0 | 0.0 |
| Switch tacrolimus for nintedanib | 5.6 | 0.0 | 10.0 | 0.0 |
| Switch tacrolimus for nintedanib + high dose prednisone | 5.6 | 0.0 | 10.0 | 0.0 |
| **Reduce dose of ILD treatment, n (%)** | **2** | **0** | **0** | **2** |
| Nintedanib | 50.0 | 0.0 | 0.0 | 50.0 |
| Low dose prednisone | 50.0 | 0.0 | 0.0 | 50.0 |

CTD, connective tissue disease; DMARD, disease-modifying antirheumatic drugs; ILD, interstitial lung disease; IPF, idiopathic pulmonary fibrosis; PPF, progressive pulmonary fibrosis.

**Supplementary Table S3.** Physician-reported most important areas of improvement for drug treatment

|  |  |  | **Type of PPF** | |
| --- | --- | --- | --- | --- |
| **Patients >10%, %** | **All patients**  **(n=309)** | **IPF**  **(n=61)** | **CTD-ILD**  **(n=136)** | **Other ILD**  **(n=112)** |
| Slows disease progression | 36.6 | 37.7 | 40.4 | 31.3 |
| Improves dyspnoea | 30.7 | 27.9 | 32.4 | 30.4 |
| Improves cough | 26.2 | 26.2 | 18.4 | 35.7 |
| Improves patients’ long-term outcomes | 25.2 | 34.4 | 29.4 | 15.2 |
| Reduced frequency of acute exacerbations of ILD | 24.6 | 34.4 | 21.3 | 23.2 |
| Improves survival / reduces ILD related mortality | 22.7 | 18.0 | 26.5 | 20.5 |
| Safe long-term use | 15.9 | 11.5 | 19.1 | 14.3 |
| Maintains efficacy over time | 14.2 | 18.0 | 11.0 | 16.1 |
| Used in accordance with guidelines | 13.6 | 13.1 | 17.7 | 8.9 |
| Physician familiarity / experience | 11.0 | 9.8 | 11.8 | 10.7 |

CTD, connective tissue disease; ILD, interstitial lung disease; IPF, idiopathic pulmonary fibrosis; PPF, progressive pulmonary fibrosis.

**Supplementary Table S4. Physician-reported reasons for patients not taking medication in the last 12 months**

|  |  |  | **Type of PPF** | |
| --- | --- | --- | --- | --- |
| **Reason for not taking medication in the last 12 months, n (%)** | **All patients** | **IPF** | **CTD-ILD** | **Other ILD** |
| **n** | **18** | **2** | **11** | **5** |
| Patient does not see an improvement | 7 (38.9) | 1 (50.0) | 3 (27.3) | 3 (60.0) |
| Does not feel instant results | 7 (38.9) | 1 (50.0) | 6 (54.5) | 0 (0.0) |
| Taking medication interferes with their lifestyle | 6 (33.3) | 0 (0.0) | 4 (36.4) | 2 (40.0) |
| Has concerns or fears over taking ILD medication | 6 (33.3) | 1 (50.0) | 3 (27.3) | 2 (40.0) |
| Patient forgets to take medication | 6 (33.3) | 2 (100.0) | 3 (27.3) | 1 (20.0) |
| Does not understand the benefits of taking medication for their ILD | 4 (22.2) | 1 (50.0) | 3 (27.3) | 0 (0.0) |
| Side effects experienced | 4 (22.2) | 0 (0.0) | 3 (27.3) | 1 (20.0) |
| Cost of medication | 4 (22.2) | 1 (50.0) | 2 (18.2) | 1 (20.0) |
| Think they only need to take medication when symptoms get worse and/or when at risk of getting worse | 3 (16.7) | 1 (50.0) | 1 (9.1) | 1 (20.0) |
| Believes their ILD can be managed without treatment | 3 (16.7) | 0 (0.0) | 2 (18.2) | 1 (20.0) |
| Thinks medication is not needed for their ILD | 2 (11.1) | 0 (0.0) | 1 (9.1) | 1 (20.0) |
| Does not like to be reliant on their medication | 2 (11.1) | 0 (0.0) | 1 (9.1) | 1 (20.0) |
| Not a high priority compared to other medications | 2 (11.1) | 0 (0.0) | 2 (18.2) | 0 (0.0) |
| None of the above | 2 (11.1) | 0 (0.0) | 1 (9.1) | 1 (20.0) |

ILD, interstitial lung disease

**Supplementary Table S5.** Patient-reported treatment adherence

|  |  | | **Type of PPF** | |
| --- | --- | --- | --- | --- |
|  | **All patients** | **IPF** | **CTD-ILD** | **Other ILDs** |
| **Medication(s) taken at a different time than advised by doctor, n (%)** | **53** | **7** | **23** | **23** |
| Never | 41.5 | 14.3 | 56.5 | 34.8 |
| Rarely | 37.7 | 85.7 | 26.1 | 34.8 |
| Sometimes | 17.0 | 0.0 | 13.0 | 26.1 |
| I have not been advised to take my medication(s) at a certain time | 3.8 | 0.0 | 4.4 | 4.3 |
| **More medication taken than advised by doctor, n (%)** | **53** | **7** | **23** | **23** |
| Never | 71.7 | 57.1 | 78.3 | 69.6 |
| Rarely | 24.5 | 42.9 | 17.4 | 26.1 |
| Sometimes | 3.8 | 0.0 | 4.3 | 4.3 |
| **Less medication taken than advised by doctor, n (%)** | **53** | **7** | **23** | **23** |
| Never | 66.0 | 42.9 | 73.9 | 65.2 |
| Rarely | 26.4 | 57.1 | 17.4 | 26.1 |
| Sometimes | 7.6 | 0.0 | 8.7 | 8.7 |
| **Forget to take medication, n (%)** | **53** | **7** | **23** | **23** |
| Never | 41.5 | 28.6 | 52.2 | 34.8 |
| Rarely | 39.6 | 57.1 | 30.4 | 43.5 |
| Sometimes | 18.9 | 14.3 | 17.4 | 21.7 |
| **Skip dose of medication(s) because patient thinks it is not working, n (%)** | **53** | **7** | **23** | **23** |
| Never | 69.8 | 57.1 | 69.6 | 73.9 |
| Rarely | 22.6 | 42.9 | 21.7 | 17.4 |
| Sometimes | 7.6 | 0.0 | 8.7 | 8.7 |
| **Skip dose of medication(s), or take less of it, because patient does not think they need it, n (%)** | **53** | **7** | **23** | **23** |
| Never | 71.7 | 85.7 | 73.9 | 65.2 |
| Rarely | 18.9 | 14.3 | 13.1 | 26.1 |
| Sometimes | 9.4 | 0.0 | 13.0 | 8.7 |
| **Miss taking medication(s), or take it at a different time than advised by doctor, because patient is not in their usual routine, n (%)** | **53** | **7** | **23** | **23** |
| Never | 47.2 | 42.9 | 52.2 | 43.5 |
| Rarely | 41.5 | 57.1 | 30.4 | 47.8 |
| Sometimes | 11.3 | 0.0 | 17.4 | 8.7 |
| **Skip taking medication(s), or take less of it, because of the cost, n (%)** | **53** | **7** | **23** | **23** |
| Never | 75.5 | 57.1 | 78.3 | 78.3 |
| Rarely | 20.7 | 42.9 | 17.4 | 17.4 |
| Sometimes | 3.8 | 0.0 | 4.3 | 4.3 |
| **Skip taking medication(s), or take less of it, because of side effects, n (%)** | **53** | **7** | **23** | **23** |
| Never | 67.9 | 57.1 | 69.6 | 69.6 |
| Rarely | 24.5 | 28.6 | 21.7 | 26.1 |
| Sometimes | 5.7 | 14.3 | 4.4 | 4.3 |
| Often | 1.9 | 0.0 | 4.3 | 0.0 |
| **Skip taking medication(s) because patient does not want other people to see them taking it, n (%)** | **53** | **7** | **23** | **23** |
| Never | 71.7 | 71.4 | 73.9 | 69.6 |
| Rarely | 24.5 | 28.6 | 21.7 | 26.1 |
| Sometimes | 3.8 | 0.0 | 4.4 | 4.3 |
| **Confidence medication(s) taken as advised by doctor, n (%)** | **53** | **7** | **23** | **23** |
| Not at all confident | 5.7 | 14.3 | 4.3 | 4.4 |
| A little confident | 15.1 | 14.3 | 8.7 | 21.7 |
| Confident | 39.6 | 42.8 | 34.8 | 43.5 |
| Very confident | 39.6 | 28.6 | 52.2 | 30.4 |
| **Medication(s) taken as often as advised by doctor, n (%)** | **52** | **7** | **22** | **23** |
| None of the time | 3.8 | 14.3 | 0.0 | 4.4 |
| A little of the time | 1.9 | 0.0 | 0.0 | 4.3 |
| Some of the time | 5.8 | 0.0 | 9.1 | 4.3 |
| Most of the time | 40.4 | 57.1 | 40.9 | 34.8 |
| All of the time | 48.1 | 28.6 | 50.0 | 52.2 |

CTD, connective tissue disease; ILD, interstitial lung disease; IPF, idiopathic pulmonary fibrosis; PPF, progressive pulmonary fibrosis.
