# Supplementary material for: Treatment patterns and clinical profile in progressive pulmonary fibrosis: a Japanese cross-sectional survey
Source: Front Med (Lausanne). 2025 Jan 15;11:1526531. doi: 10.3389/fmed.2024.1526531 (PMC11775758; doi:10.3389/fmed.2024.1526531)
Supplement: Supplementary file 3 [file Data_Sheet_2.docx]

Supplementary Material

# National Association of Friends of Collagen Disease Advisory Board Meeting | Minutes

**National Association of Friends of Collagen Disease Advisory Board Meeting on Monday February 26, 2024.**

**Group representatives: M, O, W**

**Patients: S, A**

1. **Symptoms**
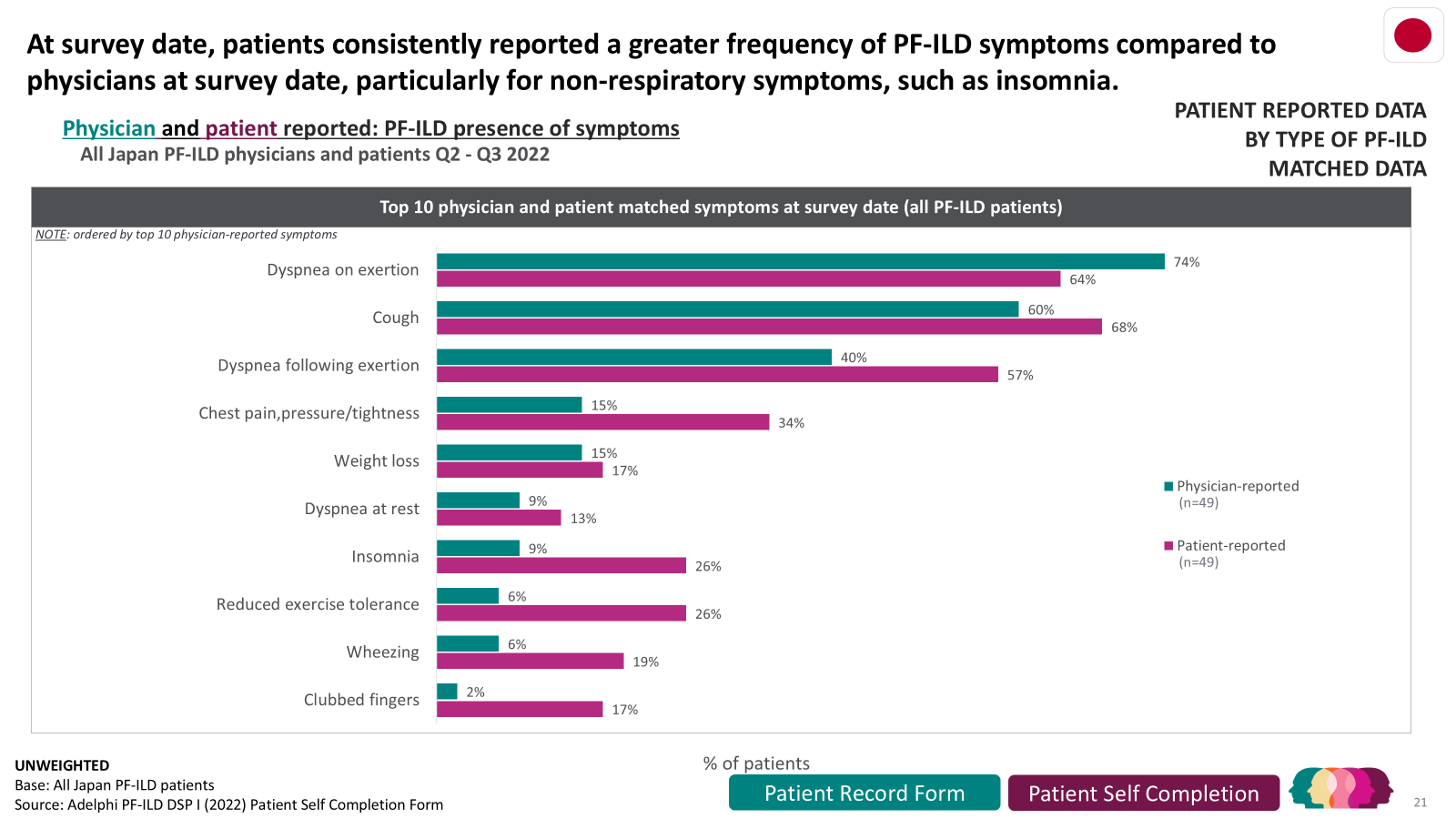

2. In general, doctors and patients perceive different symptoms, but where does this communication gap come from?
   (M) Subjective symptoms can be felt by the patient sensitively. Doctors will notice the symptoms if they are found by examination, but some symptoms are not reflected on the examination and the doctors don’t understand it.
   (A) Symptoms may not match the results of the blood test when you go to the outpatient clinic. How can I appeal to the doctor so that he understands? Some doctors are willing to listen to you (25 minutes: touching your body), but others are not. Inability to communicate is stressful. How do they understand the degree of pain? There are some parts that are difficult to understand from data alone. We tend to rely on data. I want the doctor to doubt what the patient is saying. The doctor puts a stethoscope on me.
   (S) My doctor doesn’t put a stethoscope on me.
   (M) Putting the stethoscope and taking a deep breath many times. It also gives you a sense of security if you can see it properly. Some doctors don't use stethoscopes.
   (O) I don't know if insomnia is caused by collagen disease, so I sometimes wonder whether I should say it or not. Come to think of it, there are many things that can be mentioned as symptoms. When I exercised, it became difficult to exercise. There is a discrepancy between the symptoms when they appear and when they are examined. Doctors can't look back. Patients cannot look back on their past symptoms unless their doctor tells them.
3. What do you think can close the gap?
   (W) Say what you want to say and ask doctor what you want to ask. Make notes of everything you discuss with your doctor. Some doctors only look at their computers and don't make eye contact with patients.
   (S) I wonder if it's okay to talk about this before, and I didn't say it even though I had these symptoms. If I don't have any symptoms at the time of the doctor's appointment, I forget about it. I can take notes, but it's also difficult to do that. I'm not a good patient. In particular, other people have the same symptoms of insomnia, so it is difficult to determine whether it is caused by a disease.
4. **Work Status**


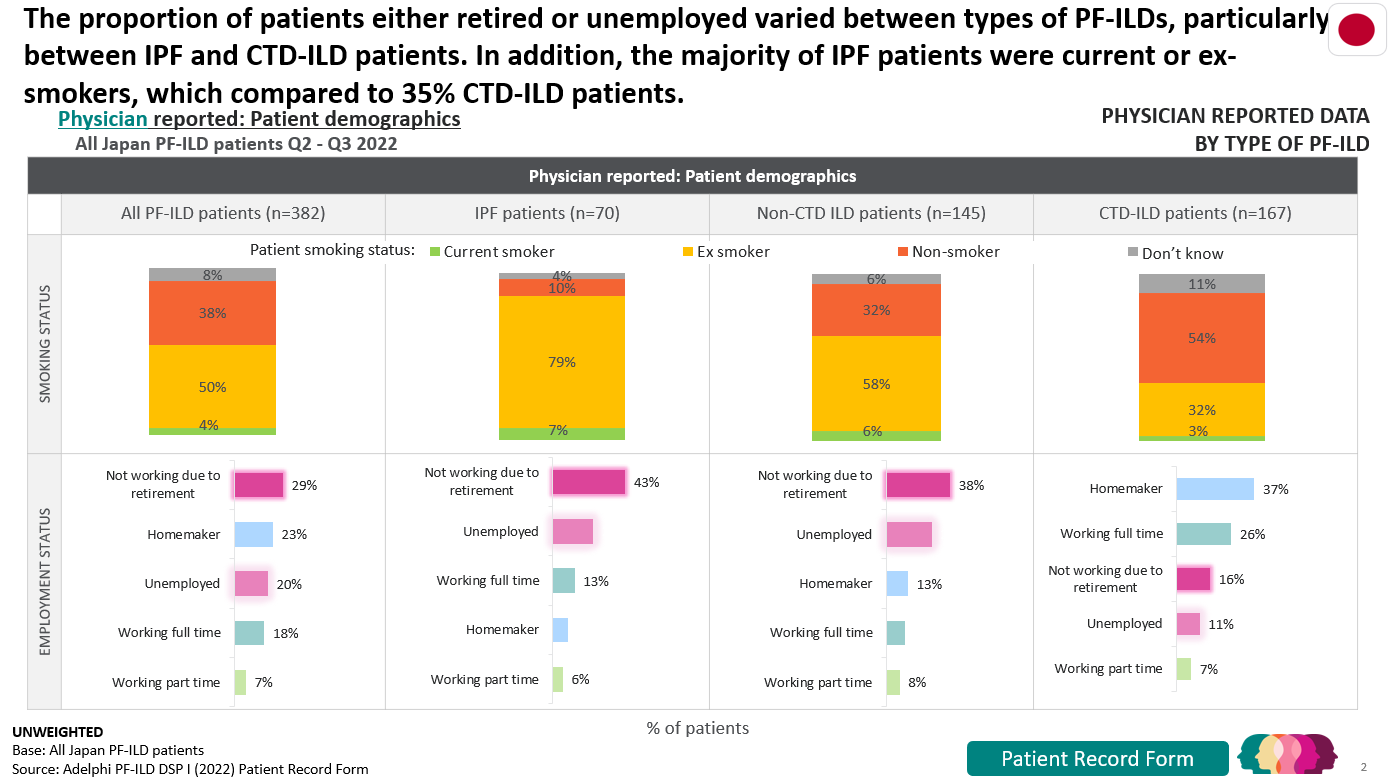


1. What are some of the difficulties you have in getting a job and working while living with interstitial lung disease?
   (W) I am self-employed and full-time. There are many people who are unemployed due to illness and leave of absence. If you are not working due to retirement, it is difficult to get back to work.
2. As a housewife, can illness be a burden when doing housework?
   (S) Housewives can use their time well. When I was working, my doctor would sometimes tell me to stop working for two or three months if I felt unwell. I was able to continue working because my workplace was understanding, but some people are not. Especially once you retire, it is difficult to come back.
3. What kind of support and system should be in place, including work and housework?
   (M) There are people who have retired due to their age, so I can't say anything. Respiratory illness is a pain during exertion, and some people may think about retiring because of commuting. It's easier to continue if you have a job where you can decide how to manage your time, such as when you have to work for a period of time. Wouldn't flextime and remote work improve the continuation of employment?
4. **Disease progression**
   1. **Doctor**


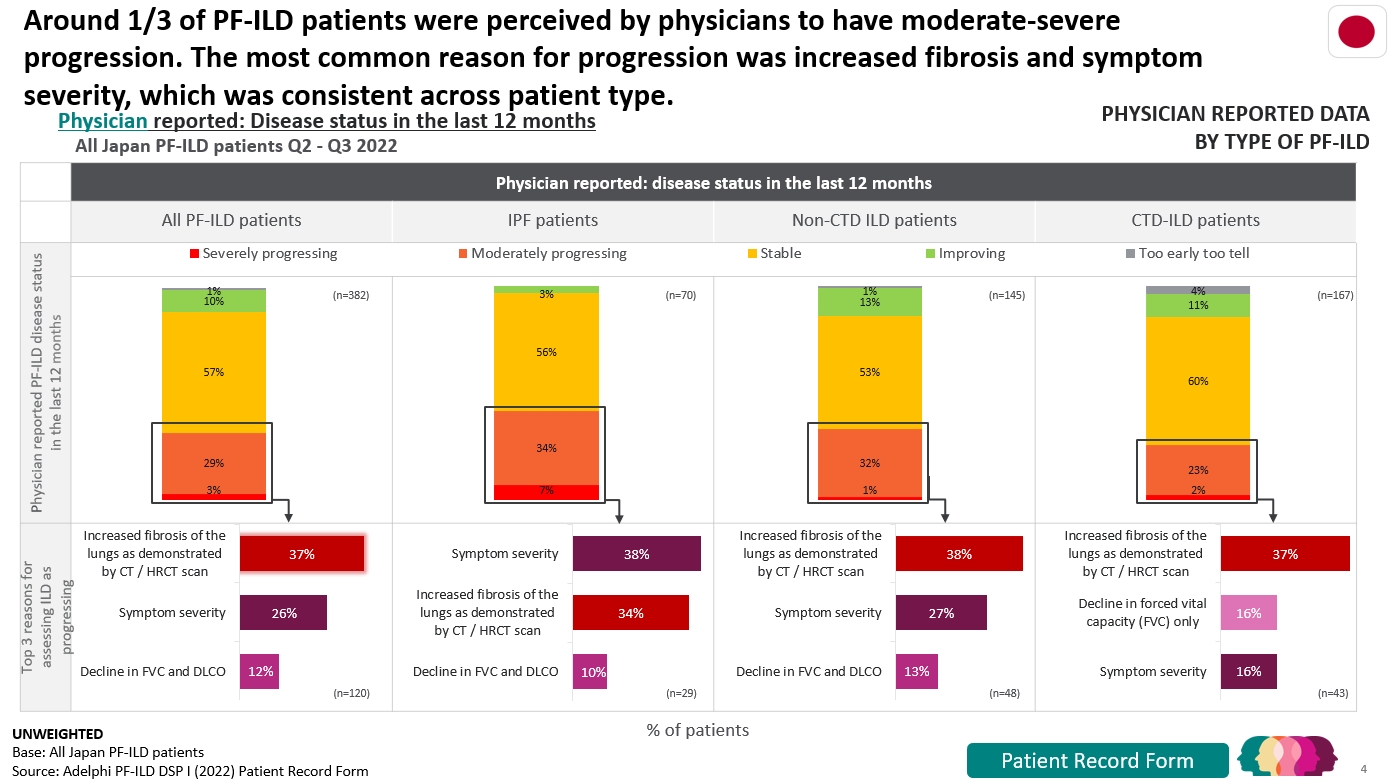


1. How and on what basis will the doctor explain the severity of the illness?
   (A) I have never talked about the severity of the disease. Symptoms vary. CT/MRI to indicate severity. I am reviewing the test results with my doctor. I can't judge whether my treatment is correct or not, but I am satisfied with it. I believe that in this state, the progression of the disease is stopped, but I also think that there is the ability to help yourself.
2. Is the doctor's explanation highly convincing? If not, what kind of explanation do you think is necessary?
   (S) There is no talk of severity. CT results look together. The doctor's explanation was, “It's getting a little worse”, so I'm not convinced. The respiratory medicine doctor and the collagen disease doctor belong to different hospitals, so they compromise with each other, and they don't give sufficient explanations, making it difficult to fully understand.
   1. **Patients**


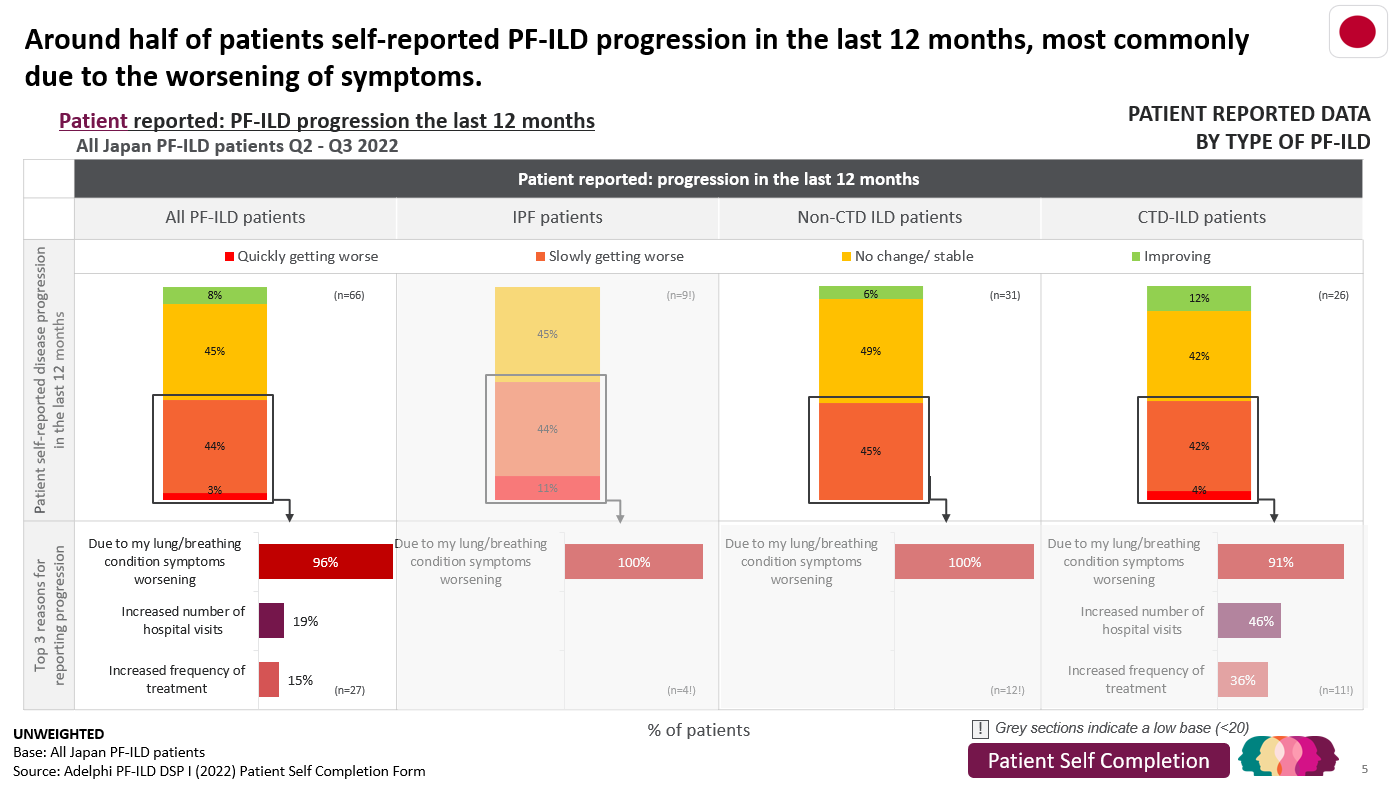


1. Decreased lung function is almost 100% of the reported reasons, but what do you think are the symptoms?
   (W) Have mild interstitial lung disease, mild myocardial infarction. I don't know if it's my heart or what's causing it. Personally, it's painful to move, but it doesn't show up in the test results. I was able to climb the stairs up to three times, but I had to rest. I’m not be able to walk distances, I’m only be able to walk slowly.
2. Doctors also use symptoms to determine progression, but are they communicating their symptoms appropriately?
   (M) I don't know if the pain is caused by the respiratory system or muscle weakness, but I can complain about the symptoms when I think about how it compares to before. It is difficult to answer whether it is now. It is easier to answer when the doctor asks if the walking speed is slowing down.
3. What do you think is necessary to correctly communicate your symptoms to your doctor?
   (A) I would like to ask the doctor how to explain it so that the doctor understands. Based on the data from the poor KL6 levels in the lungs and the results of CT scans, I think the doctor understands the pain of interstitial pneumonia. Since the numbers were very high, the treatment was intensified.
   (S) I think it's important to talk about specific things, but I wonder if the doctor will understand it, what I say to the doctor, what he wants to ask me. Since I usually teach gymnastics, I can tell doctor specifically how hard it is (not being able to exercise while talking).
4. **Treatment adherence**


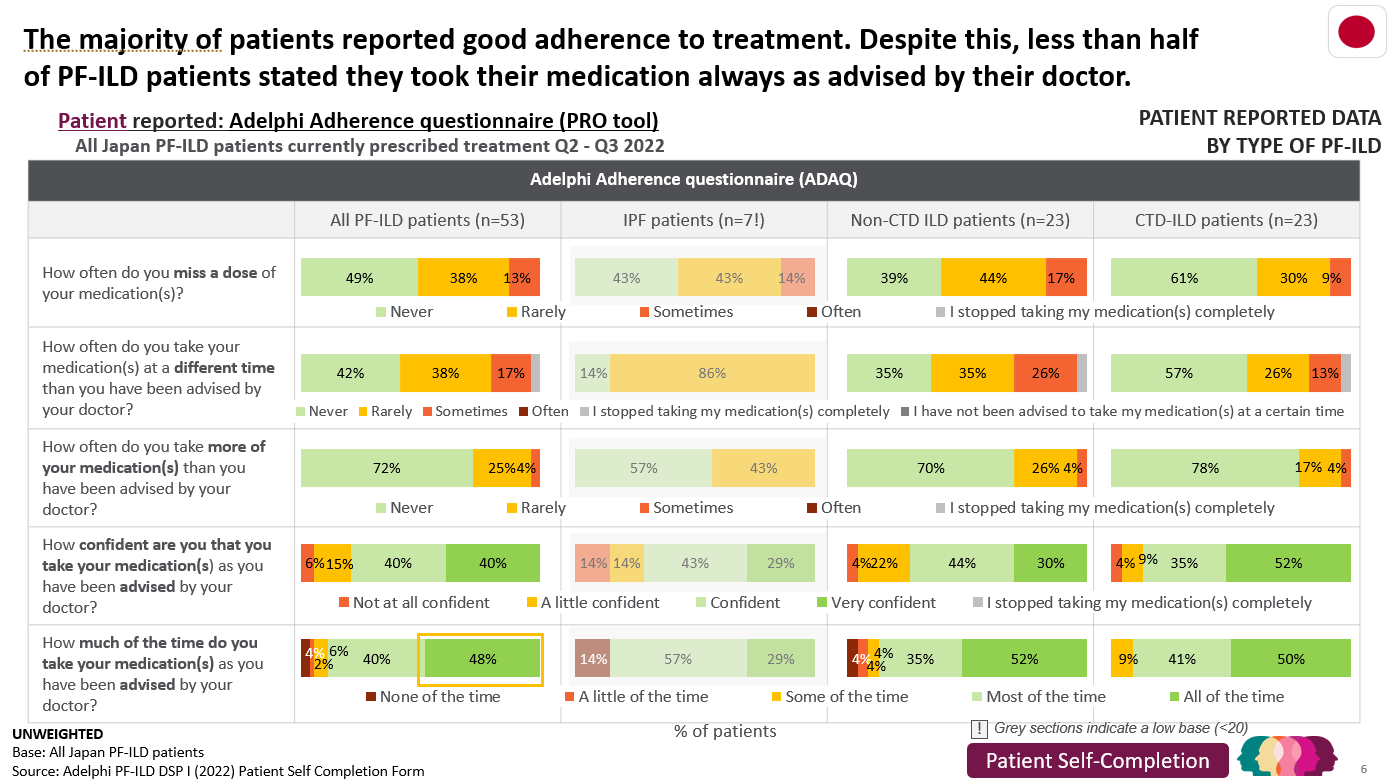


1. What do you usually do to take your medication properly?
   (W) Especially in the morning, I take 30 pills at most, and it's hard to take out the medicine. I try to take medicine after breakfast, but if I eat too much rice at the buffet, I may take it after returning to my room. It is a habit to drink immediately after every meal.
   (A) Nothing in particular, I often forget to drink at lunchtime. I have arbitrarily judged that it is not an important drug. I don't forget to drink it in the morning and evening because it is a habit and they are immunosuppressants. Morning, noon, and night, they are packaged together.
2. What is the background of good adherence and motivation for taking it?
   (O) I think I should take it with a drug for collagen disease. If I forget to take the medicine for collagen disease, I will not be able to move or it will hurt, so I can immediately know that I have forgotten to take it. If I forget to take it at night, I will be in trouble in the morning, so never forget to take it. I forget to take it during the day because it is not an important medicine (not prednin and not have effect on pain and movement), so I don't forget to take it in the morning and evening.
3. What support and services do you think can improve adherence?
   (S) As for medicine, I tell the doctor that I forgot to take lunch and have it distributed in the morning and evening. I have come to experience that if I forget, I will be in trouble, and I will not forget. I make it a habit and don't forget to take other medicines. When the medicine changes, the doctor does not explain why it has changed or what kind of medicine it is. I would like doctor to explain when doctor changes to a new drug. There is an explanation from the pharmacist, but I also want an explanation from the doctor.
   (M) Since I have collagen disease, I am taking steroids, so I don't forget to take it. When there was a medicine that was difficult to swallow and there was a large pill, I had the medicine changed to make it easier to swallow. I was told to take iron pills in the morning, but it was difficult to drink because I drank black tea in the morning, and I was able to take it at night. During the day, I was easily affected by meals, so I asked doctor to change it to another time. Talk to the doctor properly. Explaining the effects of the drug and what happens when you stop taking it may lead to improved adherence.
