# Supplementary figures and images for: Treatment patterns and clinical profile in progressive pulmonary fibrosis: a Japanese cross-sectional survey
Source: Front Med (Lausanne). 2025 Jan 15;11:1526531. doi: 10.3389/fmed.2024.1526531 (PMC11775758; doi:10.3389/fmed.2024.1526531)

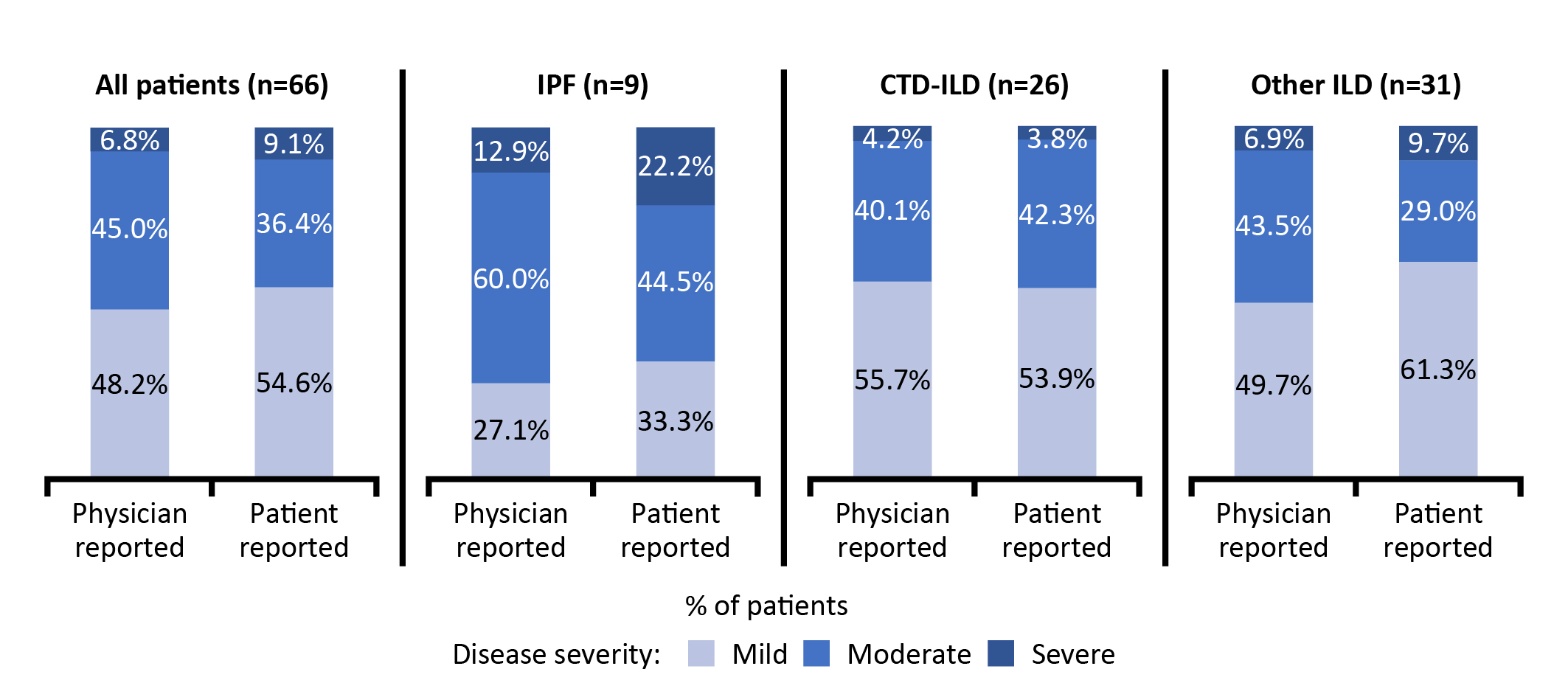

Supplement: Supplementary file 4 [file Image_1.jpeg]
